# Sirtuin 1-dependent regulation of high mobility box 1 in hypoxia–reoxygenated brain microvascular endothelial cells: roles in neuronal amyloidogenesis

**DOI:** 10.1038/s41419-020-03293-0

**Published:** 2020-12-14

**Authors:** Young-Sun Lee, Ji-Young Choi, Sakulrat Mankhong, Sohee Moon, Sujin Kim, Young Ho Koh, Ji-Hye Kim, Ju-Hee Kang

**Affiliations:** 1 Department of Pharmacology, College of Medicine, Inha University, Incheon, 22212 Republic of Korea; 2 Hypoxia-related Disease Research Center, College of Medicine, Inha University, Incheon, 22212 Republic of Korea; 3 Division of Brain Diseases, Center for Biomedical Sciences, Korea National Institute of Health, Chungcheongbuk-do, 28159 Republic of Korea; 4 Department of Kinesiology, Inha University, Incheon, 22212 Republic of Korea; 5 Department of Emergency Medicine, Inha University Hospital, Incheon, 22332 Republic of Korea

**Keywords:** Mechanisms of disease, Cellular neuroscience

## Abstract

Hypoxia–reperfusion injury is one of the major risk factors for neurodegeneration. However, it is unclear whether ischaemic damage in brain microvascular endothelial cells plays roles in neurodegeneration, particularly in the amyloidogenic changes contributing to the development of Alzheimer’s disease (AD) pathologies. Therefore, we investigated the roles of hypoxia–reoxygenation (H/R)-induced release of high mobility group box protein 1 (HMGB1), a risk molecule for AD pathogenesis in the ischaemic damaged brain, from human brain microvascular endothelial cells (HBMVECs) in neuronal amyloid-beta (Aβ) production. H/R increased nuclear–cytosolic translocation and secretion of HMGB1 in HBMVECs, along with increased permeability and HMGB1-dependent p-c-Jun activation. In addition, H/R increased the expression of Sirtuin 1 (Sirt1), coincident with an increase of intracellular Sirt1–HMGB1 binding in HBMVECs. H/R increased the acetylation of HMGB1 and extracellular secretion, which was significantly inhibited by Sirt1 overexpression. Furthermore, Sirt1 contributed to autophagy-mediated endogenous HMGB1 degradation. More importantly, treatment of neuronal cells with conditioned medium from H/R-stimulated HBMVECs (H/R-CM) activated their amyloidogenic pathways. The neuronal amyloidogenic changes (i.e. increased levels of extracellular Aβ40 and Aβ42) by H/R-CM from HBMVECs were further increased by Sirt1 inhibition, which was significantly suppressed by neutralization of the HMGB1 in H/R-CM. Collectively, our results suggest that HMGB1 derived from H/R-stimulated HBMVECs contributes to amyloidogenic pathways in neurons playing roles in the pathogenesis of AD, which are regulated by endothelial Sirt1.

## Introduction

Ischaemic brain damage as an important risk factor predisposing to dementia, can induce the accumulation of a key pathogenic molecule of Alzheimer’s disease (AD), amyloid-beta (Aβ)^1,2^ and contribute to the onset and progression of the disease^3^. In the central nervous system (CNS), microvascular endothelial cells composing the blood–brain barrier (BBB) act in maintaining CNS homeostasis and neuronal function^4^. Ischaemic damage significantly deteriorates the functions of the BBB including disruption of brain microvascular endothelial tight junctions and increased permeability^5^. Hypoxia–reoxygenation (H/R) by rapid reperfusion to resolve hypoxic damage is often associated with an exacerbation of microvascular injury that leads to an increase of diffusion and fluid filtration across the tissues^6^. BBB dysfunction following H/R injury is associated with neuroinflammation characterized by activation of glial cells and Aβ deposition^7^. Therefore, endothelial-cell-derived pathogenic factors that trigger Aβ deposition might be critical in neurodegeneration after a stroke.

AD pathologies including amyloid plaque are frequently observed in the brains of patients with post-stroke dementia, who have a 2- to 6-fold higher long-term mortality rate than stroke patients without dementia^8–10^. Therefore, elucidation of the molecular mechanisms underlying AD-related pathologies after a stroke is critical to prevent post-stroke dementia. Although studies have suggested that H/R injury should be considered as an important pathogenic factor in the development of dementia^7,8^ the specific roles of H/R-mediated molecules in Aβ accumulation have not been fully elucidated. Aβ40 and Aβ42, as primary components of amyloid plaques, are generated from amyloid precursor protein (APP) by β- and γ-secretase^11^ β-Secretase (BACE) cleaves APP to release a ~100 kDa derivative, soluble APP fragment (sAPPβ). A C-terminal fragment of APP (CTFβ) is subsequently cleaved by γ-secretase to generate Aβ, which is amyloidogenic and more prone to produce neuronal damage in the brain of patients with AD^12^. Cerebral H/R injury can induce both parenchymal and vascular endothelial injury; however, little is known about the direct roles of endothelial-cell-derived molecules in neuronal amyloidogenesis following H/R.

High mobility group box 1 (HMGB1) is a ubiquitous, non-histone DNA-binding nuclear protein that can be released into extracellular space from necrotic or damaged cells^13–15^. In hypoxia–reperfusion injury, HMGB1 is released rapidly and plays a major role in the activation of proinflammatory pathways that contribute to neurodegeneratioin^16,17^. HMGB1 in the CNS also acts as an inducer of neurite degeneration, BBB disruption, neuroimmune activities and neuronal death^17–19^. The level of HMGB1 in the cerebrospinal fluid of patients with early stages of AD pathogenesis is higher than in cognitively normal elderly subjects^17^. The release of HMGB1 is regulated by translocation from the nucleus to the cytosol with post-translational modifications such as acetylation^20,21^.

The acetylation of HMGB1 is regulated by acetyltransferases and deacetylases in several types of cells^20–22^. Sirtuin 1 (Sirt1) is a deacetylase regulating various cellular functions through the NAD^+^-dependent deacetylation of various substrates, including HMGB1. Sirt1 serves to increase autophagy^23^, which is an important mechanism in the pathogenesis of AD^24^. Furthermore, activation of Sirt1 showed protective effects against the pathogenesis of AD^25,26^. Therefore, we hypothesized that HMGB1 in human brain microvascular endothelial cells (HBMVECs) might act in AD pathogenesis under conditions of H/R injury, and that endothelial Sirt1 might regulate the HMGB1-mediated neuronal toxicity. To test this hypothesis, we investigated whether HMGB1 from HBMVECs under H/R conditions would contribute to neuronal amyloidogenesis—the major pathogenic mechanism of AD—and whether endothelial Sirt1 would regulate HMGB1-mediated amyloidogenesis.

## Results

### Permeability of HBMVECs is increased by H/R

**Fig. 1:**
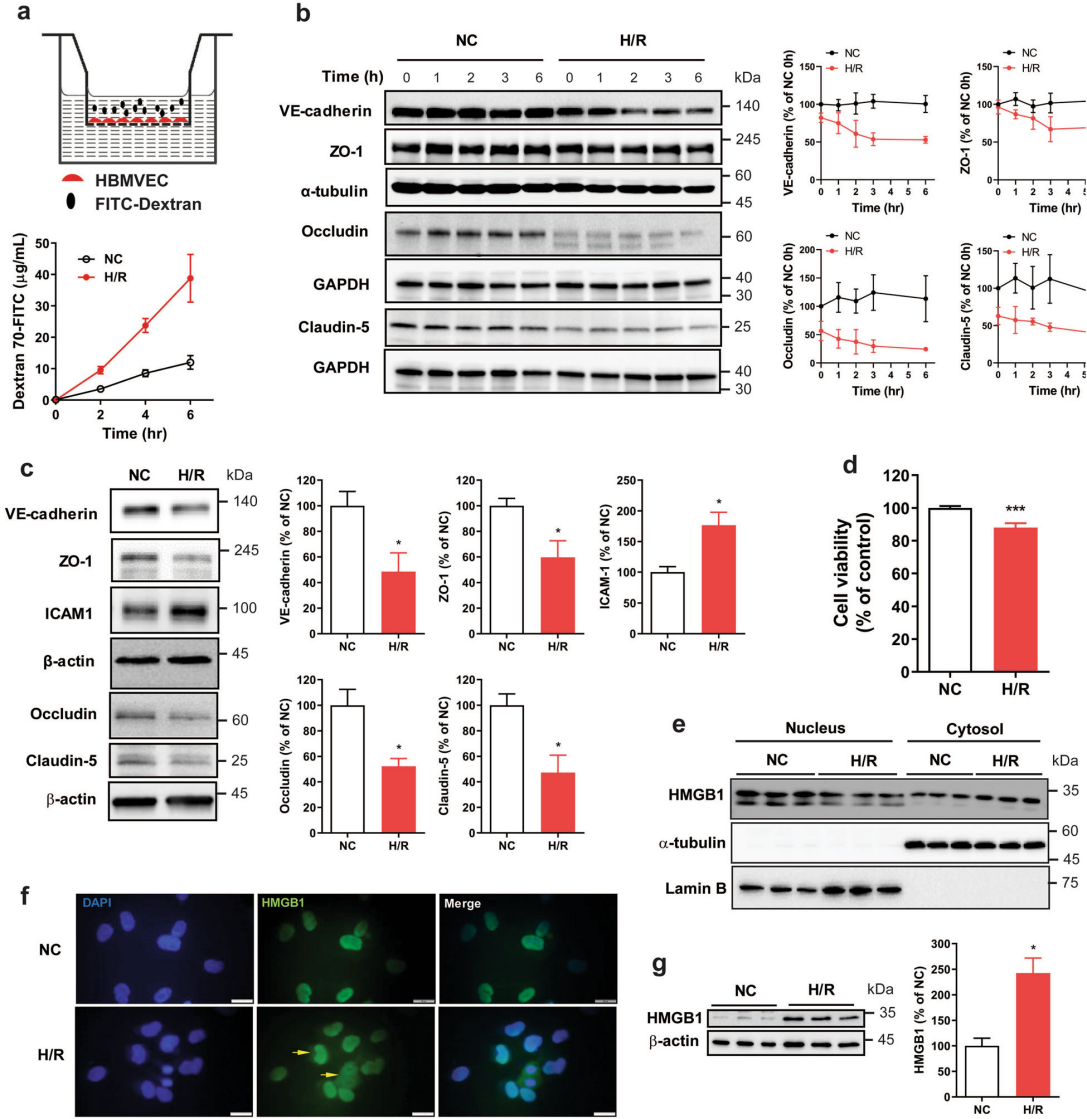
H/R increased endothelial permeability and induced extracellular secretion of HMGB1 in HBMVECs. **a, b** HBMVECs were exposed to hypoxia/glucose deprivation for 1 h followed by reoxygenation (H/R) in complete medium for the indicated times. The infiltrated FITC-dextran level in the abluminal side (**a** *n* = 6) and the expression levels of the junctional proteins VE-cadherin (*n* = 6), ZO-1 (*n* = 6), Occludin (*n* = 3) and Claudin-5 (*n* = 3) (**b**) during H/R for 6 h were measured; α-tubulin or GAPDH was used as a loading control. Representative blots are presented, and each graphs indicate the change in expression level relative to the level at 0 hr of normoxic control (NC) group, and vertical bars indicate mean ± SD. **c** Expression levels of VE-cadherin (*n* = 5), ZO-1 (*n* = 5), ICAM1 (*n* = 5), Occludin (*n* = 6) and Caludin-5 (*n* = 6) in HBMVECs with 24-h H/R were analysed by western blot analysis. β-Actin was used as a loading control. Representative blots are presented. Each bar graphs indicate the relative expression level of each protein to that of NC (mean ± SD; *p < 0.05 versus NC by unpaired *t*-test). **d** Cell viability of HBMVECs exposed to 24-h H/R was measured by MTT assay. Bar graphs indicate the mean ± SD. ***p < 0.001 versus NC. **e** Levels of HMGB1 prot**e**in in the nucleus and cytosol were analysed by western blotting. Lamin B and α-tubulin were used as loading controls for nuclear and cytosolic proteins, respectively. **f** HBMVECs with or without H/R treatment were fixed in paraformaldehyde and stained with an anti-HMGB1 antibody. Arrows indicate the cytosolic HMGB1 in cells treated with H/R for 2 h; not observed in NC. Horizontal white bars indicates 50 μm. **g** Levels of extracellular HMGB1 protein in CM from HBMVECs with or without 24-h H/R exposure were measured by western blotting; β-actin was used as a loading control (*n* = 4-5, mean ± SD; *p < 0.05 versus NC by unpaired *t-*test). Representative blots are presented.

We initially assessed the endothelial permeability of H/R-stimulated HBMVECs using fluorescein isothiocyanate (FITC)-tagged dextran as a tracer. The permeability of FITC-dextran was significantly increased at 2, 4, and 6 h after H/R in HBMVECs compared with normoxic controls without significant cell death (Fig. 1a). Concurrently, the expression of junctional molecules was decreased (Fig. 1b, c), while the expression of intercellular adhesion molecule 1 (ICAM1), an antagonist of junctions^27^ was increased after H/R in HBMVECs (Fig. 1c). Prolonged H/R (24 h) decreased the cell viability of HBMVECs (Fig. 1d), indicating that H/R induced endothelial barrier dysfunction prior to any significant endothelial cell death.

### H/R induces secretion of endothelial HMGB1

HMGB1 is a danger molecule that associates with neuronal ischemic damage and increased endothelial cell permeability and inflammation^28^. When we examined the subcellular localization and secretion of HMGB1, H/R significantly decreased nuclear HMGB1 expression while it increased the cytosolic HMGB1 expression significantly in HBMVECs (Fig. 1e). Consistent with this, immunocytochemical analysis showed that HMGB1 was localized in the cytosol of HBMVECs at 2 h after H/R, while it was restricted to the nuclei of normoxic control cells (Fig. 1f). In addition, prolonged exposure of HBMVECs to H/R (24 h) drastically increased the level of extracellular HMGB1 in the culture medium (Fig. 1g).

### H/R-induced activation of c-jun in HBMVECs

**Fig. 2:**
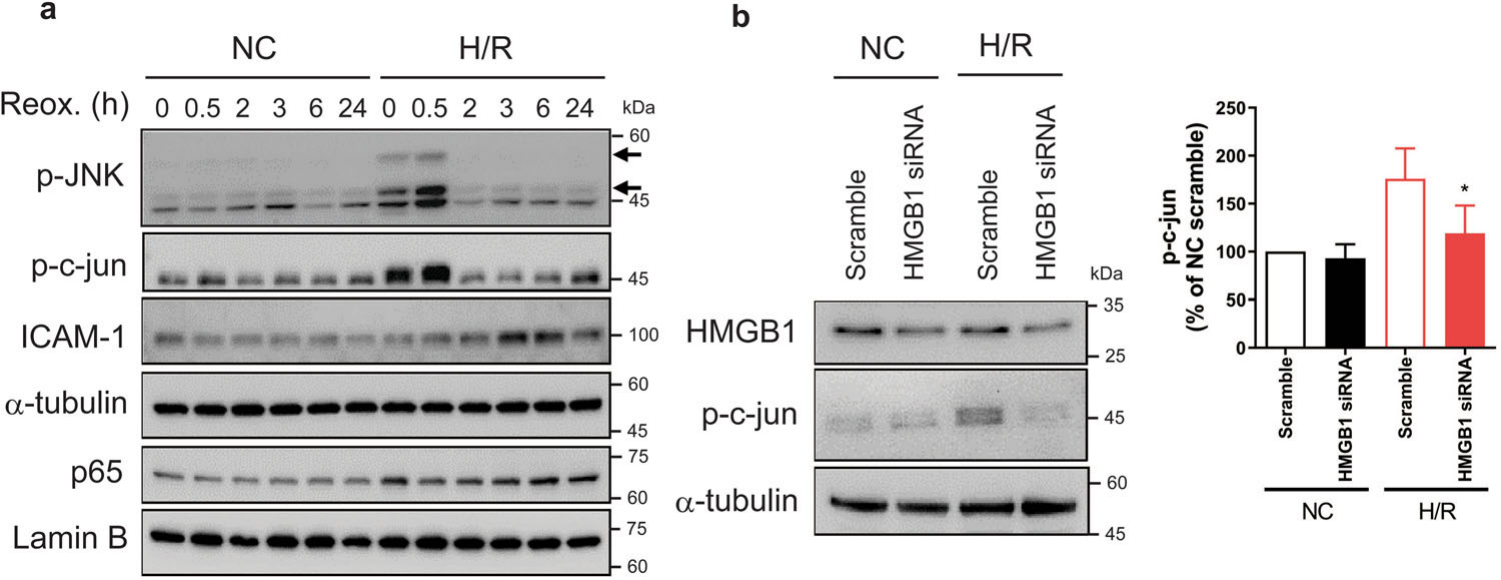
H/R activated JNK signalling and HMGB1 regulated c-Jun activation in HBMVECs. **a** HBMVECs were exposed to H/R for the indicated times. The expression levels of phosphorylated JNK (p-JNK(T183/Y185), arrows) and phosphorylated c-jun in total lysate, ICAM1 in the cytosolic fraction, and the translocation of p65 in nuclear fractions were analysed; α-tubulin and lamin B were used as loading controls. Representative blots from triplicated experiments are presented. **b** HBMVECs were transfected with scrambled siRNA (scramble) or siRNA targeting the HMGB1 gene (HMGB1 siRNA). After 24 h of transfection, HBMVECs were exposed to 24-h H/R or normoxia, and the level of intracellular p-c-Jun was analysed by western blotting; α-tubulin was used as a loading control, and representative blots are presented. Bar graph indicate the relative expression level of p-c-jun when compared with NC cells transfected scrambled siRNA (*n* = 4, mean ± SD; *p < 0.05 versus H/R scrambled siRNA group by unpaired *t-*test).

To evaluate whether HMGB1 secreted by H/R would affect inflammation in endothelial cells, we tested the time course of nuclear factor kappa-B (NF-κB) activation following H/R. Hypoxia/glucose deprivation for 1 h without reoxygenation increased the nuclear translocation of p65, a subunit of NF-κB, which was maintained until 24 h after reoxygenation (Fig. 2a). H/R increased the expression of ICAM1, a target of NF-κB, from 3 h after reoxygenation. Next, we evaluated the activation time course of the JNK/c-jun pathway involved in H/R-associated inflammation^29–32^. The basal expression of p-JNK (T183/Y185) in normoxic controls was negligible, while the expressions of p-JNK and p-c-jun were increased after only 1 h of hypoxia/glucose deprivation without reoxygenation, which was further increased by reoxygenation (Fig. 2a). Interestingly, we found that p-c-Jun showed reinduction at 24 h after H/R. Therefore, we tested whether the reinduction of p-c-jun was caused by the action of HMGB1. When we inhibited the expression of HMGB1 by short interfering (si)RNA treatment, the H/R-induced increase of p-c-jun expression was reduced significantly (Fig. 2b).

### H/R-induced secretion of HMGB1 is regulated by Sirt1

**Fig. 3:**
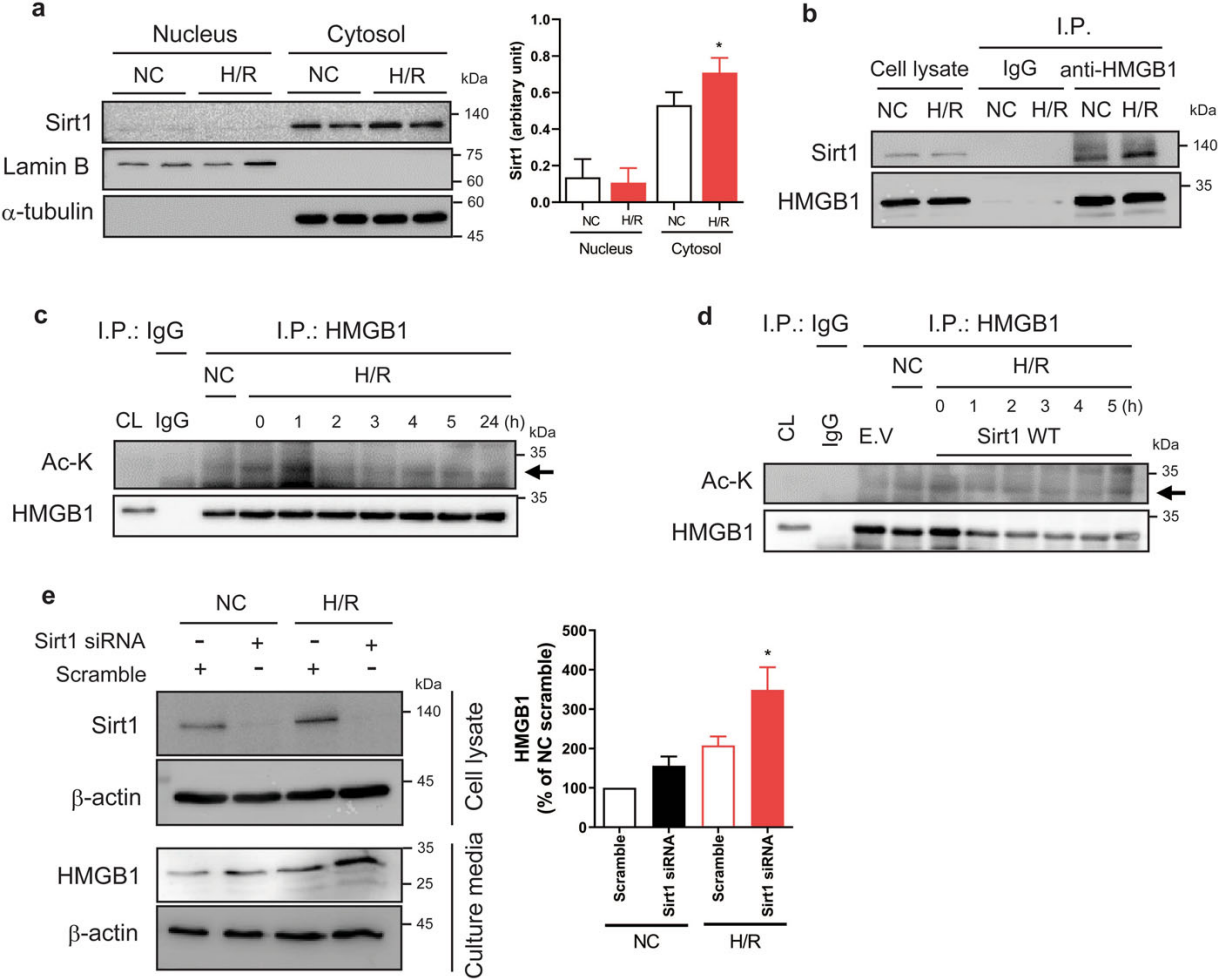
H/R induced a Sirt1-dependent acetylation of HMGB1. **a** The expression levels of Sirt1 in nuclear and cytosolic fractions were analysed in HBMVECs with exposure to NC or H/R treatments for 6 h; α-tubulin and lamin B were used as loading controls. Representative blots are presented. Bar graph indicate the level of Sirt1 in nucleus and cytosol under normoxic (NC) or hypoxia–reoxygenation (H/R) condition (*n* = 4, mean ± SD; *p < 0.05 versus cytosolic level of NC group by unpaired *t-*test). **b** HBMVECs were exposed to 6-h H/R, and 500 μg aliquots of cell lysates were immunoprecipitated (IP) with anti-HMGB1 antibody (2 μg/sample), and then protein binding was detected by western blotting using anti-Sirt1 and anti-HMGB1 antibodies. **c, d** HBMVECs (**c**) or Sirt1-overexpressing HBMVECs (**d**) were treated with H/R for 1 h and reoxygenated for the indicated times. Cell lysates (CLs) were IP with an anti-HMGB1 antibody and then acetyl-lysine (Ac-K) or HMGB1 were detected by western blot. **e** HBMVECs transfected with scrambled siRNA or Sirt1 siRNA were exposed to 24-h H/R. Sirt1 in CLs or extracellular HMGB1 was analysed by western blotting; β-actin was used as a loading control. Representative blots from are presented (*n* = 3, mean ± SD; *p < 0.05 versus H/R treated with scrambled siRNA by unpaired *t-*test).

In HBMVECs, we found that Sirt1 expression was higher in the cytosol than in the nucleus. In the cytosolic fraction, the expression of Sirt1 was significantly increased by H/R compared with normoxic conditions (Fig. 3a). As the subcellular localization of HMGB1 is regulated by acetylation^21^, we investigated whether the acetylation of HMGB1 would be regulated by Sirt1 in H/R-stimulated HBMVECs. Initially, we tested whether Sirt1 interacted with HMGB1. We found basal interaction with HMGB1 with Sirt1 in HBMVECs, which was increased by H/R (Fig. 3b). Given that the translocation of nuclear HMGB1 into the cytosol is dependent on the acetylation of lysine residues^33^, we investigated the expression of acetylated lysine (Ac-K) in HMGB1 after H/R. We found that the levels of Ac-K in immunoprecipitated HMGB1 was slightly increased in hypoxic conditions, which was further augmented by reoxygenation for 1 h and then rapidly returned to baseline (Fig. 3c). When we overexpressed the wild-type (WT) *SIRT1*, the Ac-K in HMGB1 was not induced by H/R in HBMVECs. Interestingly, Sirt1 overexpression reduced the cellular level of HMGB1 (Fig. 3d). H/R increased the endogenous expression of Sirt1 in HBMVECs, along with increases in extracellular HMGB1. Furthermore, the increased extracellular level of HMGB1 induced by H/R treatment was further increased by Sirt1 siRNA transfection (Fig. 3e).

### Destabilization of HMGB1 by Sirt1

**Fig. 4:**
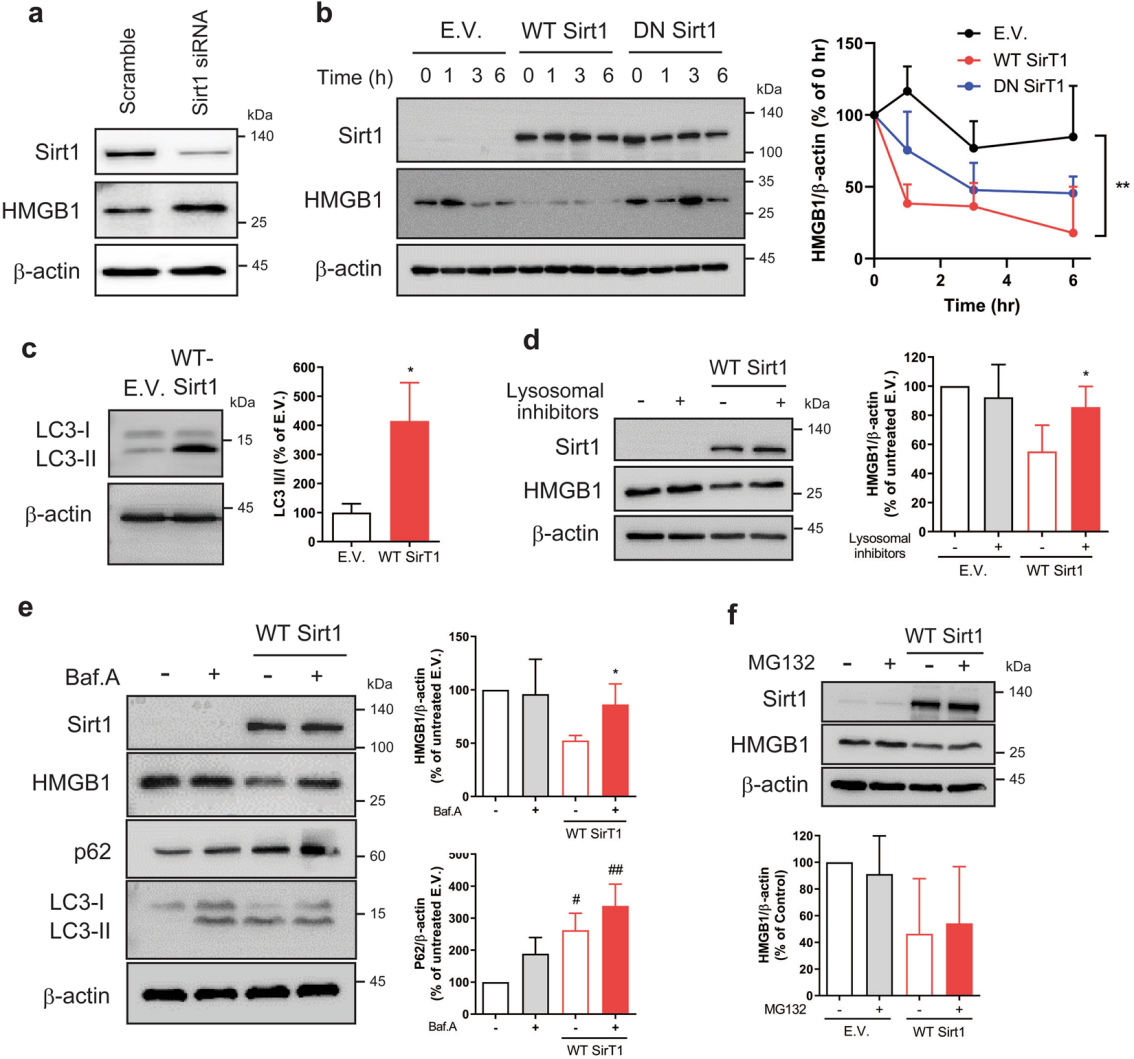
Sirt1 reduced the protein stability of intracellular HMGB1 via an autophagolysosomal pathway. **a** HBMVECs were transfected with scrambled siRNA or Sirt1 siRNA for 24 h, and the levels of Sirt1 and HMGB1 in cell lysates were analysed by western blotting; β-actin was used as a loading control. **b** HBMVECs were transfected with empty vector (E.V.), wild-type *SIRT1* (WT Sirt1) or dominant negative mutant *SIRT1* (DN Sirt1) plasmids, as described in the methodology. The levels of Sirt1 and HMGB1 were analysed by western blotting in the genetically modified HBMVECs with CHX treatment (100 μg/ml) for the indicated times. Representative blots are presented, and graph indicate the relative levels of HMGB1 at each time point as compared to 0 hr of each group (*n* = 3–4, mean ± SD; **p < 0.01 among groups by two-way ANOVA). **c**–**f** Using HBMVECs transfected with WT *SIRT1* for 24 h, cells were harvested and then analysed by western blotting using an anti-LC3 antibody (**c** *n* = 3, mean ± SD; *p < 0.05 versus E.V. by unpaired *t*-test). HBMVECs with WT *SIRT1* transfection were treated with lysosome inhibitors (**d** *n* = 3-4, mean ± SD; *p < 0.05 versus WT Sirt1 without lysosomal inhibitors by unpaired *t-*test), bafilomycin A1 (Baf.A, **e** *n* = 3, mean ± SD; *p < 0.05 versus WT Sirt1 without Baf.A by unpaired *t*-test; ^#^*p* < 0.05, ^##^*p* < 0.01 versus withou*t* treatment by one-sample *t*-test^)^, or MG132 (**f** *n* = 3, mean ± SD), and the expression levels HMGB1 were analysed. In Baf.A-treated cells, the levels of p62, and LC3 were further analysed by western blotting (**e**). β-Actin was used as a loading control. All blots are representatives from repeated experiments.

We found that Sirt1 regulated the protein level of endogenous HMGB1 in HBMVECs (Figs. 3d and 4a). Therefore, we analysed the effect of Sirt1 on HMGB1 protein stability using a cycloheximide (CHX) chasing assay and found that HMGB1 levels in HBMVECs overexpressing WT *SIRT1* decreased more rapidly and profoundly compared with dominant negative (DN) mutant *SIRT1* or empty vector control (Fig. 4b). Sirt1 acts as an inducer of autophagy^23^, and we found that WT *SIRT1* overexpression in HBMVECs-induced expression of the autophagosomal marker, LC3-II (Fig. 4c). Then, we tested whether the decreased stability of HMGB1 protein produced by Sirt1 was mediated by autophagy using a lysosome inhibitor cocktail (E64, leupeptin and aprotinin) or an inhibitor of autophagosome/lysosome fusion (bafilomycin A1). The endogenous HMGB1 protein was reduced by WT *SIRT1* overexpression, which was partially restored by lysosome inhibitors or bafilomycin A (Fig. 4d, e). Accompanying HMGB1 downregulation, Sirt1 overexpression increased the levels of p62 and the LC3-II/LC3-I ratio (Fig. 4e), indicating that Sirt1 overexpression induced autophagy. However, a proteasome inhibitor (MG132) could not restore the HMGB1 protein level in Sirt1-overexpressing HBMVECs (Fig. 4f).

### Activation of neuronal amyloidogenic pathways by conditioned medium from Sirt1-inhibited and H/R-stimulated HBMVECs

**Fig. 5:**
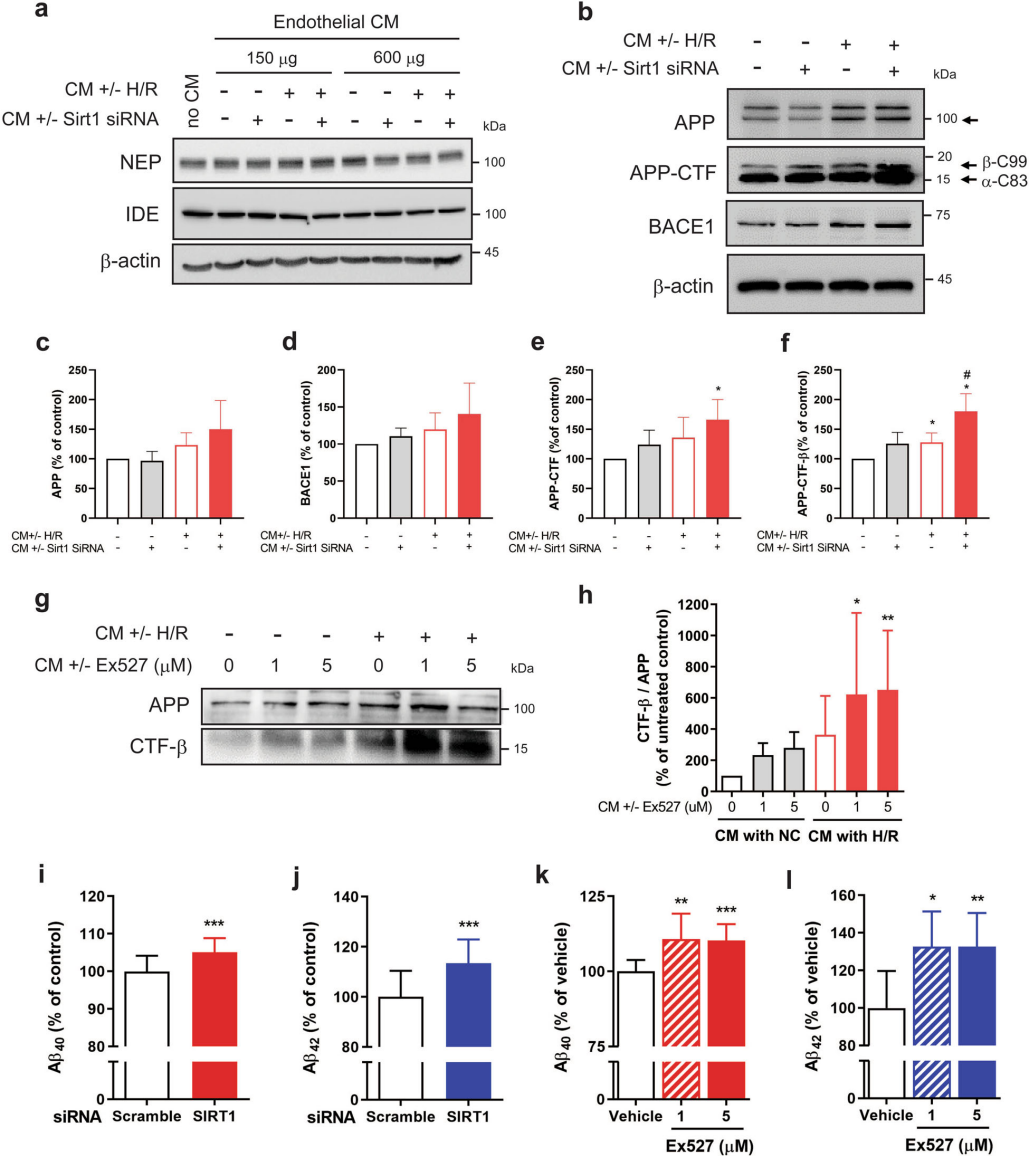
Inhibition of Sirt1 in H/R-stimulated HBMVECs increased amyloidogenic pathway in neuronal cells. **a**–**h** HBMVECs were transfected with scrambled or Sirt1 siRNA for 24 h followed by exposure to 24-h H/R or normoxia, and CM samples were harvested. SH-SY5Y cells (**a**) or H4swe cells (**b**–**h**) were cultured with HBMVEC–CM for 24 h. The levels of neprilysin (NEP) and IDE in SH-SY5Y cells were analysed by western blotting; β-actin was used as a loading control. Representative blots from duplicated experiment are presented (**a**). In H4swe cells, the expression of full-length APP (APP-N), APP-CTF (CTF-alpha, α-C83 and CTF-beta, β-C99) and BACE1 were detected by western blotting; β-actin was used as a loading control. Representative blots are presented, and bar graph indicate the semi-quantitative levels of full-length APP, APP-CTF, APP-CTFβ and BACE1, as compared to CM without H/R or Sirt1 siRNA (**b**–**f**, *n* = 3, mean ± SD *p < 0.05 versus without treatment by one-sample *t*-test, ^#^*p* < 0.05 versus H/R CM without treatment of Sirt1 siRNA by unpaired *t-*test). HBMVECs were treated with Ex527 (a Sirt1 inhibitor) at 1 μM (Ex. 1) or 5 μM (Ex. 5) for 24 h, followed by 24-h H/R or normoxia treatment. H4swe cells were cultured with the HBMVECs NC-CM or H/R-CM for 24 h. Full-length APP (APP-N) and the carboxy-terminal fragment of CTFβ were detected by western blotting. Representative blots are presented (**g**), and bar graph indicate the semi-quantitative levels of CTF-β/APP (**h** *n* = 5, mean ± SD; *p < 0.05, **p < 0.01 versus CM with normoxia without treatment of Ex527 by one-way ANOVA with multiple comparison). **i**–**l** HBMVECs were transfected with scrambled or Sirt1 siRNA (**i**, **j**) or treated with Ex527 (**k**, **l**) for 24 h followed by 24-h H/R exposure. H4swe cells were cultured with each CM for 24 h. The concentrations of Aβ40 and Aβ42 in H4swe CM were analysed using ELISA. Data are shown as means ± SD from repeated independent experiments (*n* = 9-15, *p < 0.05, **p < 0.01, ***p < 0.001 versus scrambled or vehicle control by unpaired *t-*tests).

To investigate whether factors secreted from H/R-stimulated HBMVECs directly induce neuronal amyloidogenesis, we examined amyloidogenesis and the levels of extracellular Aβ forms (Aβ40 and Aβ42) in neurons treated with conditioned medium (CM) derived from H/R-stimulated and/or Sirt1-inhibited HBMVECs. Measuring the endogenous expression of neprilysin and insulin-degrading enzyme (IDE)—proteases involved in Aβ degradation—in human neuroblastoma SH-SY5Y cells, the expression of neprilysin was reduced by CM from HBMVECs with H/R treatment or with Sirt1 siRNA transfection (Fig. 5a). Next, we tested the effects of H/R and Sirt1 regulation on amyloid precursor protein (APP) metabolism using H4swe cells. Endothelial CM from H/R-stimulated and Sirt1-inhibited cells significantly increased the level of the C-terminal fragment of APP (βC99) in H4swe cells (Fig. 5b, f), which was reproducible in an experiment using a pharmacological Sirt1 inhibitor, Ex527 (Fig. 5g, h). Consistent with these findings, the levels of extracellular Aβ40 and Aβ42 from H4swe cells were significantly increased by CM from HBMVECs with H/R and Sirt1 inhibition, compared with CM from HBMVECs treated with H/R only (Fig. 5i–l).

### Extracellular HMGB1 in CM from HBMVECs subjected to H/R increases the production of neuronal Aβ

**Fig. 6:**
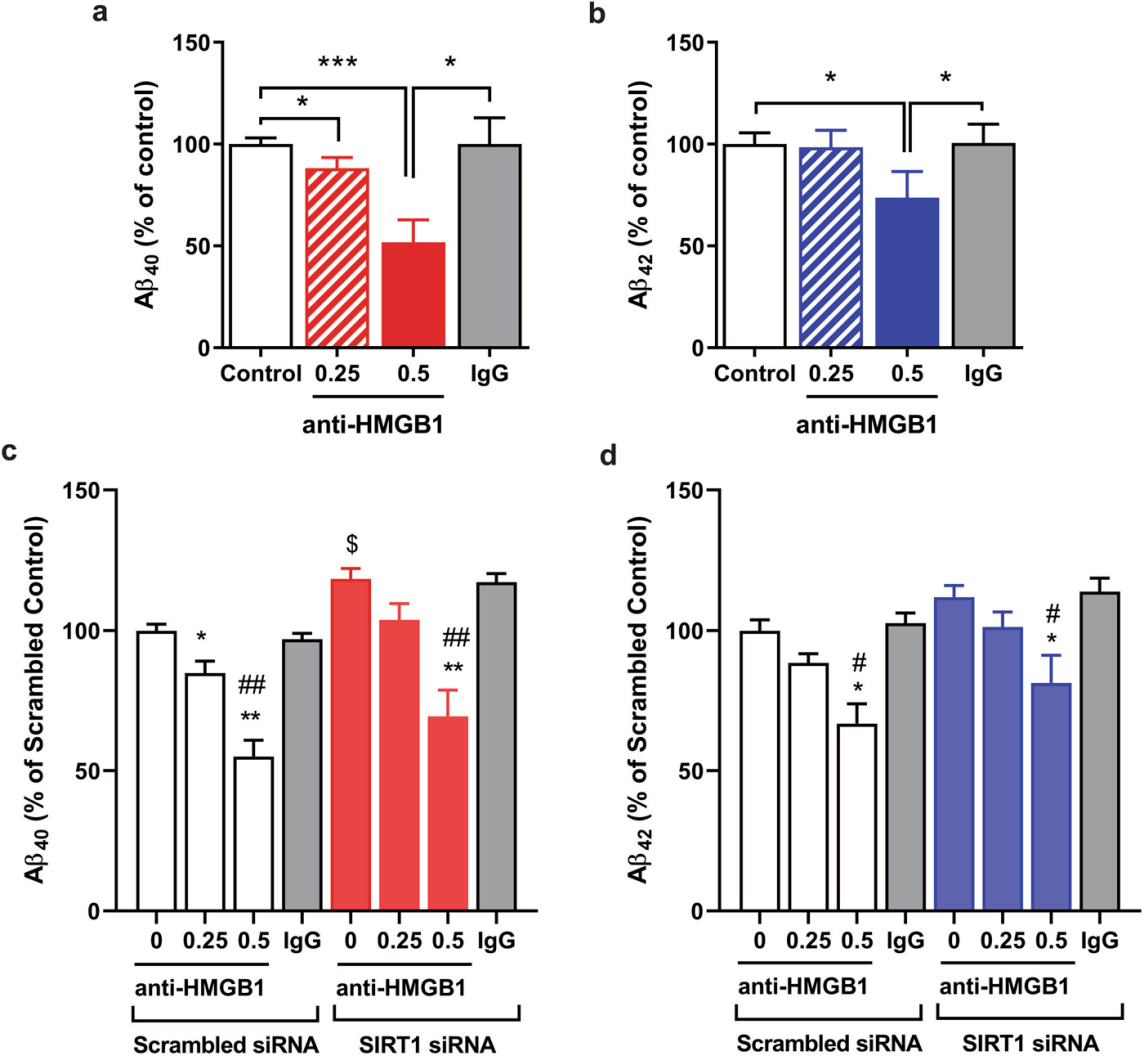
Effects of anti-HMGB1 neutralizing antibody on the levels of extracellular Aβ released from H4swe cells treated with H/R-conditioned medium (CM) from HBMVECs. HBMVECs were exposed to H/R for 1 h and reoxygenated for 24 h, and CM collected. This was treated with anti-HMGB1 antibody (0.25 or 0.5 μg/ml), and then H4swe cells were cultured with H/R CM with or without neutralization for 24 h. The concentrations of Aβ40 (**a**) and Aβ42 (**b**) in CM were analysed by ELISA. Data are presented as the mean ± SD from repeated independent experiments (*n* = 3–4). *p < 0.05, ***p < 0.001 by unpaired *t*-tests. HBMVECs with or without Sirt1 silencing were exposed to 24-h H/R, and then H4swe cells were treated with the endothelial CM with or without neutralization of HMGB1. The levels of Aβ40 (**c**) and Aβ42 (**d**) in H4swe CM were analysed by ELISA. Data are presented as mean ± SD from repeated independent experiments (*n* = 3–4). *p < 0.05, **p < 0.01 versus vehicle control^; #^*p* < 0.05, ^##^*p* < 0.01 versus IgG, ^$^*p* < 0.05 versus the control scrambled siRNA group by unpaired *t*-tests.

To confirm whether extracellular HMGB1 in CM from H/R-stimulated HBMVECs would directly induce the production of Aβ40 and Aβ42 in neurons, we tested the effect of an anti-HMGB1 neutralizing antibody. Using CM from H/R-stimulated HBMVECs with or without immunoneutralization of HMGB1, we measured the extracellular levels of Aβ40 and Aβ42 secreted by H4swe cells treated with CM. The extracellular levels of both Aβ40 and Aβ42 were significantly and dose-dependently reduced by the immunoneutralization treatment, but not by CM from control IgG-treated cells (Fig. 6a, b). In addition, this immunoneutralization of Aβ production of the amyloidogenic CM was also observed in cells treated with CM from HBMVECs with both H/R and Sirt1 siRNA transfection. Sirt1 inhibition slightly attenuated the neutralizing effects of the anti-HMGB1 antibody (Fig. 6c, d).

## Discussion

The integrity of the BBB is disrupted in the brain following ischaemic damage as well as in the brains of subjects with AD, leading to reduced levels of junctional proteins. Here, we found that the H/R-induced activation of JNK pathway was significantly inhibited by HMGB1 inhibition. Because the increased endothelial permeability followed by JNK activation is associated with neuronal death and neuroinflammation after ischaemic brain injury^29,30^, the HMGB1-dependent JNK activation in endothelial cells might partially contribute to BBB disruption and ischaemic brain damage. However, p65-dependent inflammation induced by H/R might be independent from the effect of secreted HMGB1. Given that brain endothelial cells have paracrine activity^34,35^, secreted endothelial HMGB1 following nuclear–cytosolic translocation after H/R might play crucial roles in H/R-mediated neuronal damage. The HMGB1 protein has multiple functions, which depend on its location: nucleus, cytosol, or extracellular^33^. Numerous studies have shown that post-translational modification of HMGB1 contributes to its export from the nucleus to the cytosol and active extracellular secretion, which plays diverse pathologic roles in diseases^19,22,36–39^.

The acetylation of HMGB1 is involved in subcellular localization, and forced hyperacetylation of HMGB1 in resting macrophages induces its translocation and secretion^22^. Furthermore, contributions of extracellular HMGB1 in pathogenesis have been reported for various diseases including sepsis, arthritis, ischaemia–reperfusion injury, and neurodegeneration^15–17,36,39^. As demonstrated in Fig. 3, we found that rapid acetylation of lysine residues in HMGB1 induced by H/R was dependent on Sirt1, which might be via direct binding with cytosolic Sirt1. Furthermore, Sirt1 inhibition contributed to the increased extracellular levels of HMGB1. Thus, it appears that H/R treatment induced extracellular secretion of HMGB1 via the acetylation of HMGB1 that was regulated by Sirt1 in HBMVECs.

Sirt1 deacetylates multiple histone and non-histone substrates^40^, and hypoxia increases Sirt1 expression^41^. However, the roles of endothelial Sirt1 in acetylation and release of HMGB1 associated with neuronal amyloidosis have not been elucidated. We found that the direct binding of cytosolic Sirt1 following H/R in HBMVECs was associated with the inhibition of HMGB1 release. Using chemical inhibitors of protein stability, we found that Sirt1-dependent decrease in protein stability of HMGB1 was partially attributed to the autophagolysosomal pathway (Fig. 4). Cytosolic HMGB1 can be secreted actively through an unconventional lysosomal pathway in an acetylation-dependent manner^42,43^. Moreover, Sirt1 contributes to the activation of autophagy through deacetylation autophagy-related genes^23^. Therefore, our results suggest that upregulation of cytosolic Sirt1 by H/R in HBMVECs induces deacetylation of cytosolic HMGB1, which might partially reinforce its lysosomal degradation, although roles of Sirt1 in unconventional secretion pathways of HMGB1 cannot be excluded^42^.

More importantly, we observed that CM from HBMVECs under H/R conditions activated amyloidogenic APP metabolism in neurons, which was further aggravated by Sirt1 inhibition (Fig. 5). Although the additional effect of Sirt1 inhibition plus H/R treatment on Aβ-degrading enzymes was minimal, Sirt1 inhibition augmented Aβ production by H/R-related extracellular factors, indicating that Sirt1 inhibition modified the H/R-stimulated CM to generate neuronal Aβ. Among possible extracellular factors arising from H/R treatment that induce amyloidogenesis, we focused on the role of endothelial HMGB1. The significant inhibition of Aβ production from H4swe cells by HMGB1 neutralization of H/R-stimulated endothelial CM indicated that endothelial cells secreted amyloidogenic factors under H/R conditions including—at least in part—HMGB1 (Fig. 6). It has been widely accepted that accumulation of extracellular amyloidogenic Aβ is a key factor in AD pathogenesis. Therefore, Sirt1-mediated inhibition of HMGB1 release, or neutralization of HMGB1 from endothelial cells might be a target for the prevention of post-stroke neurodegeneration. A study in neurons suggested that regulation of Sirt1 might be a therapeutic target in treating AD via increases in autophagy and antiamyloid activity^25^. Furthermore, HMGB1 inhibits microglial Aβ clearance^44,45^. Therefore, Sirt1 activation after a stroke may be a strategy to prevent subsequent neurodegeneration by inhibition of both neuronal and endothelial amyloidogenic pathways as well as HMGB1-mediated neuroinflammation.

Our study had several limitations. First, our results have not been confirmed in animal models testing whether endothelium-specific Sirt1 might regulate HMGB1 release and amyloidogenesis after H/R injury. Nevertheless, our results have clearly demonstrated the roles of endothelial Sirt1-mediated HMGB1 regulation in neuronal amyloidosis and imply that endothelial injury by vascular risk factors might be an important target to prevent amyloid pathogenesis in the development of AD, in which Sirt1 activity in both endothelia and neurons might be important. Second, we did not measure amyloid production using primary neurons. However, our study has implicated the roles of endothelial-cell-derived HMGB1 in neuronal amyloidogenesis, which needs to be further evaluated in animal models and/or primary neurons. Finally, our results do not exclude the involvement of HMGB1 acetylation by other deacetylases or acetylases besides Sirt1, because HMGB1 is a potential target for these^46,47^. Therefore, regulation of HMGB1 acetylation/secretion in brain endothelial cells by other mechanisms should be evaluated further.

In conclusion, H/R-stimulated HBMVECs released HMGB1 during Sirt1-dependent regulation of acetylation and autophagosomal degradation of HMGB1, which might regulate the level of extracellular HMGB1. Importantly, the Sirt1-dependent HMGB1 release from HBMVECs after H/R treatment contributed to neuronal amyloidogenesis. Therefore, Sirt1-dependent endothelial HMGB1 secretion in patients following a stroke might be a target to prevent progression to AD, although further in vivo and clinical studies are needed to confirm this.

## Materials and methods

### Cell culture

HBMVECs (ACBRI 376, Cell Systems, Kirkland, WA, USA) were cultured in M199 medium (Invitrogen, Carlsbad, CA, USA) with 20% heat-inactivated foetal bovine serum (FBS, HyClone, GE Healthcare Bio-Sciences, Pittsburgh, PA, USA), 5 U/ml heparin, 3 ng/ml basic fibroblast growth factor (Merck Millipore, Temecula, CA, USA), 100 U/ml penicillin, and 100 μg/ml streptomycin at 37 °C in a humidified atmosphere (95% air and 5% CO_2_). HBMVECs were seeded and grown in type-1 collagen-coated flasks. H4swe cells, a human neuroblastoma cell line containing Swedish FAD mutant APP695, were used as described^24^. These were grown in Dulbecco’s modified Eagle’s medium (DMEM; Invitrogen) with 10% FBS, 25 mM glucose, 400 μg/ml G418, 100 U/ml penicillin and 100 μg/ml streptomycin at 37 °C. SH-SY5Y cells (ATCC, Manassas, VA) were cultured in DMEM supplemented with 10% FBS, 25 mM glucose, 100 U/ml penicillin and 100 μg/ml streptomycin at 37 °C in a humidified atmosphere of 95% air and 5% CO_2_.

### Hypoxia with glucose deprivation (H/R) and reoxygenation

H/R experiments were carried out in a purpose-built hypoxia glove-box chamber (InVivO2 400, Ruskinn Technologies, Pencoed, UK) maintained at 5% CO_2_ at 37 °C. The O_2_ concentration was monitored constantly using an O_2_ sensor. HBMVECs were treated with hypoxia (0.5% O_2_) and glucose deprivation for 1 h in Earle’s balanced salt solution (EBSS; 116 mM NaCl, 5.37 mM KCL, 0.8 mM MgSO_4_.7H_2_O, 1.8 mM CaCl_2_, 1.01 mM NAH_2_PO_4_, 26.19 mM NaHCO_3_), and then the cells were restored to 37 °C with fresh M199 medium (with 2% FBS) in a humidified atmosphere of 95% air and 5% CO_2_ for the indicated durations.

### Measurement of endothelial permeability

Endothelial permeability was assessed by measuring fluorescein isothiocyanate (FITC)-tagged 70 kDa dextran (Sigma–Aldrich, St. Louis, MO) flux across monolayers of cultured HBMVECs. Endothelial cells were seeded on top of transwell chambers and grown to confluence. After hypoxia for 1 h in EBSS, 2% FBS M199 with FITC-dextran was changed to the upper (luminal) chamber. Relative fluorescence (485 nm excitation/535 nm emission) of FITC-dextran in medium from the lower (abluminal) chamber was determined at 0, 2, 4 and 6 h of incubation by collection of 100 μl triplicate aliquots using a fluorescence plate reader (PerkinElmer, Waltham, MA, USA).

### Treatment of CM from H/R-stimulated HBMVECs

Samples of CM from HBMVECs after oxygen restoration were collected at 24 h after reoxygenation, centrifuged at 500 × *g* for 10 min at 4 °C to pellet the cellular debris, and supernatants were used immediately. H4swe or SH-SY5Y cells were exposed to CM from HBMVECs with or without H/R for 24 h. Aβ40 and Aβ42 levels in the CM of H4swe cells were determined with commercial enzyme-linked immunosorbent assay (ELISA) kits following the manufacturer’s instructions (Invitrogen).

### Western blotting

Total cell lysates were prepared using radioimmunoprecipitation assay (RIPA) buffer or mammalian protein extraction buffer (GE Healthcare Bio-Sciences) containing both a protease inhibitors and phosphatase inhibitors cocktail (Sigma–Aldrich). The isolated protein was electrophoresed using 10% or 15% polyacrylamide sodium dodecyl sulphate gel electrophoresis (SDS–PAGE) and transferred to polyvinylidene difluoride membranes. After 1-h blocking with 3% skim milk, membranes were incubated with primary antibodies as appropriate. Membranes were then incubated with horseradish peroxidase-conjugated goat anti-rabbit IgG or goat anti-mouse IgG antibodies. Signals were detected with enhanced chemiluminescence detection kits (Merck Millipore) and analysed using a Chemi doc System (Bio-Rad, Hercules, CA, USA).

### Immunocytochemistry

HBMVECs were fixed in 4% paraformaldehyde, incubated with a permeation buffer (Thermo Fisher Scientific, Waltham, MA, USA), blocked, and then incubated with a rabbit anti-HMGB1 antibody (1:100; Abcam, Cambridge, UK). FITC-conjugated goat anti-rabbit IgG (Santa Cruz Biotechnology, Dallas, TX, USA 1:200) was used as a secondary antibody; 4′,6-diamidino-2-phenylindole (Sigma–Aldrich) solution was used as a nuclear counterstain. Fluorescence was captured using a laser scanning confocal fluorescence microscope (LSM 700, Carl Zeiss, Oberkochen, Germany).

### Analysis of cell viability

The viability of HBMVECs was measured using a 3-(4,5-dimethylthiazol-2-yl)-2,5-diphenyltetrazolium (MTT) assay as described^48^. Briefly, MTT (0.5 mg/ml) was added to each well followed by incubation at 37 °C for 2–3 h. After removal of supernatant, formazan crystals were lysed with DMSO at 37 °C for 10 min. Optical density was measured at 570 nm using a microplate reader and the results are expressed as a percentage of control cells.

### Transfection with small interfering (si)RNAs

The siRNAs were purchased from GenePharma Co. Ltd. (Shanghai, P. R. China). The sequences targeting the HMGB1 gene were 5′–GAU GCA GCU UAU ACG AAA UTT–3′ and 5′–AUU UCG UAU AAG CUG CAU CTT–3′. For Sirt1 silencing, siRNAs with sequences of 5′–GCU GGC CUA AUA GAG UGG CAA–3′ and 5′–UUG CCA CUC UAU UAG GCC AGC–3′ were used. Transfections of HMGB1 or Sirt1 siRNAs were performed using RNAi Max (Invitrogen) according to the manufacturer’s protocol. Plasmids encoding Flag-tagged WT or dominant negative (DN) mutant H363Y *SIRT1* were donations from Dr Michael Greenberg (Addgene; http://n2t.net/addgene:1791;RRID:Addgene1791). The empty construct pcDNA3.1 plasmid was transfected as a control. Transfection of the plasmids was conducted using Lipofectamine 2000 (Invitrogen) following the manufacturer’s protocol.

### Immunoprecipitation

HBMVEC lysates (500 μg protein) were incubated overnight with anti-HMGB1 (Abcam) of 2 μg per sample at 4 °C. Twenty microlitres of agarose conjugate suspension (GE Healthcare Life Sciences, Buckinghamshire, UK) was added to the samples, and mixtures were incubated at 4 °C with rotation for 3 h. After brief centrifugation, pellets were washed with RIPA buffer. Pellets were resuspended in sample buffer and boiled for 10 min. These samples were used for SDS–PAGE and immunoblotting with anti-HMGB1, anti-Sirt1 or anti-acetylated-lysine antibodies (Abcam).

### Ethics statement

Our study did not use animal or human samples or data, therefore this study did not require ethical approval.

### Statistical analysis

Data are presented as the mean ± SD. Differences between groups for cell viability, band densities from western blots or levels of Aβ after independently multiple experiments were analysed using unpaired Student’s *t*-tests, one-sample *t*-test or ANOVA with multiple comparison, as appropriate (Prism v 6.0, Graphpad Software, San Diego, CA); *p* < 0.05 was accepted as significant.

## References

[1] Hardy J (2009). The amyloid hypothesis for Alzheimer’s disease: a critical reappraisal. J. Neurochem..

[2] Bell RD, Zlokovic BV (2009). Neurovascular mechanisms and blood-brain barrier disorder in Alzheimer’s disease. Acta Neuropathol..

[3] Marchesi VT (2011). Alzheimer’s dementia begins as a disease of small blood vessels, damaged by oxidative-induced inflammation and dysregulated amyloid metabolism: implications for early detection and therapy. FASEB J..

[4] Ohtsuki S (2013). Quantitative targeted absolute proteomic analysis of transporters, receptors and junction proteins for validation of human cerebral microvascular endothelial cell line hCMEC/D3 as a human blood-brain barrier model. Mol. Pharm..

[5] Sandoval KE, Witt KA (2008). Blood-brain barrier tight junction permeability and ischemic stroke. Neurobiol. Dis..

[6] Eltzschig HK, Eckle T (2011). Ischemia and reperfusion–from mechanism to translation. Nat. Med..

[7] Zlokovic BV (2011). Neurovascular pathways to neurodegeneration in Alzheimer’s disease and other disorders. Nat. Rev. Neurosci..

[8] Leys D, Henon H, Mackowiak-Cordoliani MA, Pasquier F (2005). Poststroke dementia. Lancet Neurol..

[9] Desmond DW, Moroney JT, Sano M, Stern Y (2002). Mortality in patients with dementia after ischemic stroke. Neurology.

[10] Barba R (2002). Previous and incident dementia as risk factors for mortality in stroke patients. Stroke.

[11] Thinakaran G, Koo EH (2008). Amyloid precursor protein trafficking, processing, and function. J. Biol. Chem..

[12] Murphy MP, LeVine H (2010). Alzheimer’s disease and the amyloid-beta peptide. J. Alzheimers Dis..

[13] Liu Y, Prasad R, Wilson SH (2010). HMGB1: roles in base excision repair and related function. Biochim. Biophys. Acta.

[14] Bell CW, Jiang W, Reich CF, Pisetsky DS (2006). The extracellular release of HMGB1 during apoptotic cell death. Am. J. Physiol. Cell Physiol..

[15] Scaffidi P, Misteli T, Bianchi ME (2002). Release of chromatin protein HMGB1 by necrotic cells triggers inflammation. Nature.

[16] Ye Y (2019). The role of high mobility group box 1 in ischemic stroke. Front. Cell Neurosci..

[17] Festoff BW, Sajja RK, van Dreden P, Cucullo L (2016). HMGB1 and thrombin mediate the blood-brain barrier dysfunction acting as biomarkers of neuroinflammation and progression to neurodegeneration in Alzheimer’s disease. J. Neuroinflammation.

[18] Fujita K (2016). HMGB1, a pathogenic molecule that induces neurite degeneration via TLR4-MARCKS, is a potential therapeutic target for Alzheimer’s disease. Sci. Rep..

[19] Hei Y (2018). HMGB1 neutralization attenuates hippocampal neuronal death and cognitive impairment in rats with chronic cerebral hypoperfusion via suppressing inflammatory responses and oxidative stress. Neuroscience.

[20] Bonaldi T (2003). Monocytic cells hyperacetylate chromatin protein HMGB1 to redirect it towards secretion. EMBO J..

[21] Wang Y, Wang L, Gong Z (2019). Regulation of acetylation in high mobility group protein B1 cytosol translocation. DNA Cell Biol..

[22] Hwang JS (2015). Deacetylation-mediated interaction of SIRT1-HMGB1 improves survival in a mouse model of endotoxemia. Sci. Rep..

[23] Lee IH (2008). A role for the NAD-dependent deacetylase Sirt1 in the regulation of autophagy. Proc. Natl Acad. Sci. USA.

[24] Cho SJ (2015). SUMO1 promotes Abeta production via the modulation of autophagy. Autophagy.

[25] Anekonda TS, Reddy PH (2006). Neuronal protection by sirtuins in Alzheimer’s disease. J. Neurochem..

[26] Lalla R, Donmez G (2013). The role of sirtuins in Alzheimer’s disease. Front. Aging Neurosci..

[27] Clark PR, Manes TD, Pober JS, Kluger MS (2007). Increased ICAM-1 expression causes endothelial cell leakiness, cytoskeletal reorganization and junctional alterations. J. Invest. Dermatol..

[28] Huang W (2012). HMGB1 increases permeability of the endothelial cell monolayer via RAGE and Src family tyrosine kinase pathways. Inflammation.

[29] Nijboer CH (2010). Inhibition of the JNK/AP-1 pathway reduces neuronal death and improves behavioral outcome after neonatal hypoxic-ischemic brain injury. Brain Behav. Immun..

[30] Wang LW (2014). TNFR1-JNK signaling is the shared pathway of neuroinflammation and neurovascular damage after LPS-sensitized hypoxic-ischemic injury in the immature brain. J. Neuroinflammation.

[31] Ferrer I, Friguls B, Dalfo E, Planas AM (2003). Early modifications in the expression of mitogen-activated protein kinase (MAPK/ERK), stress-activated kinases SAPK/JNK and p38, and their phosphorylated substrates following focal cerebral ischemia. Acta Neuropathol..

[32] Shvedova M, Anfinogenova Y, Atochina-Vasserman EN, Schepetkin IA, Atochin DN (2018). c-Jun N-terminal kinases (JNKs) in myocardial and cerebral ischemia/reperfusion injury. Front. Pharm..

[33] Yang H, Wang H, Chavan SS, Andersson U (2015). High mobility group box protein 1 (HMGB1): The prototypical endogenous danger molecule. Mol. Med.

[34] Wang J (2013). Insulin-like growth factor-1 secreted by brain microvascular endothelial cells attenuates neuron injury upon ischemia. FEBS J..

[35] Yu QJ, Tao H, Wang X, Li MC (2015). Targeting brain microvascular endothelial cells: a therapeutic approach to neuroprotection against stroke. Neural Regen. Res..

[36] Andrassy M (2008). High-mobility group box-1 in ischemia-reperfusion injury of the heart. Circulation.

[37] Hreggvidsdottir HS (2012). High mobility group box protein 1 (HMGB1)-partner molecule complexes enhance cytokine production by signaling through the partner molecule receptor. Mol. Med..

[38] Wang H (1999). HMG-1 as a late mediator of endotoxin lethality in mice. Science.

[39] Harris HE, Andersson U, Pisetsky DS (2012). HMGB1: a multifunctional alarmin driving autoimmune and inflammatory disease. Nat. Rev. Rheumatol..

[40] Martinez-Redondo, P. & Vaquero, A. The diversity of histone versus nonhistone sirtuin substrates. *Genes Cancer* **4**, 148–163 (2013). 10.1177/1947601913483767 PMC3764476 24020006

[41] Chen R, Dioum EM, Hogg RT, Gerard RD, Garcia JA (2011). Hypoxia increases sirtuin 1 expression in a hypoxia-inducible factor-dependent manner. J. Biol. Chem..

[42] Gardella S (2002). The nuclear protein HMGB1 is secreted by monocytes via a non-classical, vesicle-mediated secretory pathway. EMBO Rep..

[43] Lee H (2010). Analysis of nuclear high mobility group box 1 (HMGB1)-binding proteins in colon cancer cells: clustering with proteins involved in secretion and extranuclear function. J. Proteome Res..

[44] Takata K (2004). High mobility group box protein-1 inhibits microglial Abeta clearance and enhances Abeta neurotoxicity. J. Neurosci. Res..

[45] Takata K (2012). Microglial amyloid-beta1-40 phagocytosis dysfunction is caused by high-mobility group box protein-1: implications for the pathological progression of Alzheimer’s disease. Int. J. Alzheimers Dis..

[46] Lin TB (2016). Melatonin relieves neuropathic allodynia through spinal MT2-enhanced PP2Ac and downstream HDAC4 shuttling-dependent epigenetic modification of hmgb1 transcription. J. Pineal Res.

[47] Ong SP, Lee LM, Leong YF, Ng ML, Chu JJ (2012). Dengue virus infection mediates HMGB1 release from monocytes involving PCAF acetylase complex and induces vascular leakage in endothelial cells. PLoS ONE.

[48] Oh YM (2009). Inhibition of 6-hydroxydopamine-induced endoplasmic reticulum stress by l-carnosine in SH-SY5Y cells. Neurosci. Lett..

